# Recent Developments in Isothermal Amplification Methods for the Detection of Foodborne Viruses

**DOI:** 10.3389/fmicb.2022.841875

**Published:** 2022-03-03

**Authors:** Cassandra Suther, Sloane Stoufer, Yanjiao Zhou, Matthew D. Moore

**Affiliations:** ^1^Department of Food Science, University of Massachusetts Amherst, Amherst, MA, United States; ^2^Department of Medicine, University of Connecticut Health, Farmington, CT, United States

**Keywords:** isothermal amplification, virus, foodborne virus, LAMP (loop mediated isothermal amplification), RPA (recombinase polymerase amplification), PCR

## Abstract

Foodborne and enteric viruses continue to impose a significant public health and economic burden globally. As many of these viruses are highly transmissible, the ability to detect them portably, sensitively, and rapidly is critical to reduce their spread. Although still considered a gold standard for detection of these viruses, real time polymerase chain reaction (PCR)-based technologies have limitations such as limited portability, need for extensive sample processing/extraction, and long time to result. In particular, the limitations related to the susceptibility of real time PCR methods to potential inhibitory substances present in food and environmental samples is a continuing challenge, as the need for extensive nucleic acid purification prior to their use compromises the portability and rapidity of such methods. Isothermal amplification methods have been the subject of much investigation for these viruses, as these techniques have been found to be comparable to or better than established PCR-based methods in portability, sensitivity, specificity, rapidity, and simplicity of sample processing. The purpose of this review is to survey and compare reports of these isothermal amplification methods developed for foodborne and enteric viruses, with a special focus on the performance of these methods in the presence of complex matrices.

## Introduction

Viruses are the leading cause of foodborne illness in the United States, representing about 65% of known foodborne illnesses (Vasickova et al., 2005). A variety of viruses are known to cause foodborne disease, including human norovirus, sapoviruses, enteroviruses, hepatitis A and E viruses, astroviruses, rotaviruses and adenoviruses, among others. These viruses are typically enteric, meaning they replicate in the gut and can be transmitted through the fecal-oral route (Greening and Cannon, 2016). Numerous foods have been implicated in foodborne virus transmission, many of which are ready-to-eat foods that are commonly consumed without cooking. In many cases, viral contamination of food occurs from food handlers and/or fecally contaminated water. Some examples of foods commonly implicated in foodborne virus outbreaks include berries, spinach, oysters and other mollusks (Shieh et al., 1999; Dubois et al., 2002; Flannery et al., 2012; Rönnqvist et al., 2014; Summa and Maunula, 2018). While norovirus is the most prevalent of these viruses, it has been estimated that norovirus, rotavirus, hepatitis A and hepatitis E viruses collectively kill over 620,000 people worldwide each year (Moore and Jaykus, 2017b), highlighting the importance of controlling their spread.

Unlike many bacterial and fungal foodborne pathogens, an enrichment step or growth of pathogen for higher detection, is not feasible for routine detection of foodborne viruses. Further, the high transmissibility and low infectious dose of many foodborne viruses means that portable, rapid, and sensitive detection of viruses is important for controlling their spread. Ligand-based detection methods, including enzyme-linked immunosorbent assay (ELISA) and immunochromatography, can offer portability and relatively rapid results. However, these methods often lack the analytical sensitivity required for detection of such viruses in foods and the environment (Liu and Moore, 2020). Further, such methods can also have high rates of false positive or negative results depending upon the ligand used (Niizuma et al., 2013). The current gold standard for the detection and quantification of foodborne viruses is real time reverse transcriptase polymerase chain reaction (RT-qPCR). This technique often has a relatively favorable analytical sensitivity; however, traditional RT-qPCR can lack portability, is prone to food matrix-associated inhibition, and can take >1.5 h (Klein, 2002; Moore and Jaykus, 2017b).

Isothermal amplification methods have been the focus of research for several decades, as many isothermal amplification techniques offer the benefits of better portability, favorable analytical sensitivity, rapid time to result, potentially superior inhibitor tolerance, and potentially higher fidelity. In particular, the lower energy demand of isothermal techniques better enables portable detection, as often assays can be incubated in portable battery-powered devices due to the lower energy demand. As discussed below, the reaction time for these assays is also traditionally shorter in many cases, reducing the time to result and getting closer to true point-of-care testing. However, a number of challenges can still exist with these methods, in particular the requirement for upstream processing to concentrate and purify nucleic acid prior to use in the assay, the ability to increase assay throughput, and the expense of some of the reagents for some of the assays. Several isothermal techniques have shown promise for detection of foodborne and enteric viruses. Some examples of these include Recombinase Polymerase Amplification (RPA) (Daher et al., 2016), Loop-Mediated Isothermal Amplification (LAMP) (Abdullahi et al., 2015), Nucleic Acid Sequence-Based Amplification (NASBA) (Deiman et al., 2002), Rolling Circle Amplification (RCA) (Mohsen and Kool, 2016), Helicase-Dependent Amplification (HDA) (Cao et al., 2013), Transcription-Reverse Transcription Concerted Assay (TRC) (Lv et al., 2012; Medici et al., 2013), and Cross Priming Amplification (CPA) (Xu et al., 2012). In addition to portability and rapidity, these isothermal techniques have demonstrated sufficient analytical sensitivity for detecting the low viral levels at which food and environmental contamination often occurs. The purpose of this review is to highlight and discuss these techniques and progress in the development of isothermal amplification methods for foodborne viruses (Table 1), with a particular focus on the performance of these methods in complex matrices (Table 2).

**TABLE 1:** Comparison of limits of detection observed for different isothermal assays developed against foodborne and enteric viruses.

Isothermal amplification method	Vial target	Limit of detection with reaction volume	^a^Limit of detection normalized to/μL	References
***Caliciviridae* family**				
Recombinase polymerase amplification (RPA)	Norovirus	0.8–10.0 LGC/50 μL reaction	0.068 ± 0.004 LGC/μL	Moore and Jaykus, 2017a
	Norovirus	1.66 × 102 copies/μL using 50 μL reaction	166 copies/μL	Han et al., 2020
Loop-mediated isothermal amplification (LAMP)	Norovirus	102 and 103 copies/25 μL reaction	4 and 40 copies/μL	Fukuda et al., 2006
	Norovirus	22 copies/μL using 10 μL reaction	22 copies/μL	Khairuddin et al., 2017
	Norovirus	103 copies/25 μL reaction	40 copies/μL	Luo et al., 2014
	Norovirus	103 copy/20 μL reaction	50 copies/μL	Fukuda et al., 2008
	Norovirus	103 copy/20 μL reaction	50 copies/μL	Iturriza-Gómara et al., 2008
	Norovirus	4.7 × 102 copies/μL using 25 μL reaction	470 copies/μL	Jeon et al., 2017
Nucleic acid sequence-based amplification (NASBA)	Norovirus	104 PCR units/ml using 20 μL reaction	10 PCR units/μL	Greene et al., 2003
	Norovirus	0.01 PCR units using 20 μL reaction	0.0006 PCR units/μL	Lamhoujeb et al., 2009
***Picornaviridae* family**				
Recombinase polymerase amplification (RPA)	Enterovirus 71	3.767 log10 copies/50 μL reaction	0.07594 log10 copies/μL	Yin et al., 2018
	Coxsackievirus A16	0.55 TCID50/25 μL reaction	0.022 TCID50/μL	Yan et al., 2015
	Coxsackievirus A6	400 copies/50 μL reaction	8 copies/μL	Wang et al., 2017
Loop-mediated isothermal amplification (LAMP)	Human enterovirus A & B	10 genomic copies/μL using 25 μL reaction	10 genomic copies/μL	Zhao et al., 2014
	Enterovirus 71	0.01 PFU/25 μL reaction	0.0004 PFU units/μL	An et al., 2014
	Enterovirus 71	0.33 TCID50/reaction per 25 μL reaction	0.0132 TCID50/μL	Nie et al., 2011
	Coxsackievirus A16	1.58 TCID50/reaction per 25 μL reaction	0.0632 TCID50/μL	Nie et al., 2011
	Coxsackievirus A16	81 copies/reaction per 25 μL reaction	3.24 copies/μL	Yaqing et al., 2012
	Coxsackievirus B	0.1 pg RNA/12.5 μL reaction	0.008 pg RNA/μL	Monazah et al., 2017
	Coxsackievirus B	0.1 pg RNA/12.5 μL reaction	0.008 pg RNA/μL	Zeinoddini et al., 2017
	Poliovirus	400 copies/12.5 μL reaction	32 copies/μL	Arita et al., 2009
	Hepatitis A	0.4–0.8 FFU/12.5 μL reaction	0.016–0.032 FFU/μL	Yoneyama et al., 2007
Nucleic acid sequence-based amplification (NASBA)	Human enterovirus A and B	< 100 copies RNA per reaction	No volume provided	Fox et al., 2002
	Coxsackievirus B	10 pg RNA/20 μL reaction	0.5 pg RNA/μL	Zeinoddini et al., 2017
	Hepatitis A	0.4 ng of RNA/ml using 25 μL reaction volumes	0.0004 ng RNA/μL	Jean et al., 2001
***Astroviridae* family**				
Loop-mediated isothermal amplification (LAMP)	Astrovirus	3.6 copies/μL using 25 μL reaction volumes	3.6 copies/μL	Yang et al., 2014
***Adenorovirusiridae* family**				
Recombinase polymerase amplification (RPA)	Adenovirus	50 copies/50 μL reaction	1 copies/μL	Rames and Macdonald, 2019
Loop-mediated isothermal amplification (LAMP)	Adenovirus	50–100 copies/20 μL reaction	2.5–5 copies/μL	Ziros et al., 2015

*^a^Limit of detection normalized to per μL for the purposes of comparison; however, volume associated dependence of reaction cannot be dismissed.*

*LGC, log10 genomic copies; PFU, Plaque forming units; TCID50, Median Tissue Culture Infectious Dose.*

**TABLE 2:** Matrix inhibition without nucleic acid extraction when using isothermal amplification.

Isothermal amplification method	Sample Matrix	Viral target	Limit of detection with reaction volume	^a^Limit of detection normalized to/μL	References
Recombinase polymerase amplification (RPA)	20% heat treated human fecal suspensions	GII.4 New Orleans norovirus	Detected 61% of boiled stool samples	N/A	Moore and Jaykus, 2017a
	2% heat treated human fecal suspensions	Norovirus GII.4 New Orleans	Detected 58% of boiled stool samples	N/A	Moore and Jaykus, 2017a
	Simple heat-treatment of nasopharyngeal swab specimens	EV71	1.6 TCID50/20 μL reaction	0.8 TCID50/μL	Nie et al., 2012
Loop-mediated isothermal amplification (LAMP)	Sewage samples	Adenoviruses 40 and 41	Detected 93.75% (15/16) of urban sewage samples	N/A	Ziros et al., 2015
Nucleic acid sequence-based amplification (NASBA)	Raw wastewater	Hepatitis A virus	106 PFU/mL per 25 μL reaction	1,000 PFU/μL	Jean et al., 2001
	Wastewater after aerobic digestion with activated sludge	Hepatitis A virus	106 PFU/mL per 25 μL reaction	1,000 PFU/μL	Jean et al., 2001
	Wastewater after aerobic digestion and UV treatment	Hepatitis A virus	106 PFU/mL per 25 μL reaction	1,000 PFU/μL	Jean et al., 2001
	Lettuce	Hepatitis A virus	108 PFU/mL per 25 μL reaction	100,000 PFU/μL	Jean et al., 2001
	Blueberries	Hepatitis A virus	108 PFU/mL per 25 μL reaction	100,000 PFU/μL	Jean et al., 2001

*^a^Limit of detection normalized to per μL for the purposes of comparison; however, volume associated dependence of reaction cannot be dismissed.*

*LGC, log10 genomic copies; PFU, Plaque forming units.*

## Consistency Is Key: Popular Isothermal Amplification Methods

RPA, LAMP and NASBA have been the most frequently reported isothermal methods developed for foodborne viruses. For the purposes of this review, only these methods will be covered.

NASBA was first developed by Greene et al. (2003), and is designed to detect only RNA targets (Yan et al., 2014). Sometimes referred to as transcription mediated amplification (TMA), the assay is based on the reaction of two enzymes, avian myeloblastosis virus reverse transcriptase (RT), and RNase H/T7 DNA dependent RNA polymerase (DdRp), which work together to start a strand-dependent amplification reaction (Fakruddin et al., 2012). Reactions run for around 60–90 min at a constant temperature ranging from 40 to 55°C.

LAMP is a technique that was developed about two decades ago, in 2000 (Notomi et al., 2000). Since its invention, many commercial kits have emerged. LAMP employs auto-cyclizing strand displacement of genomic material using a *Bst* DNA polymerase. This process includes a set of four primer pairs, which recognize six specific sequences on the target strand (Dhama et al., 2014). This technique has been reported to be more sensitive than PCR in some cases when detecting different viruses (Parida et al., 2004, 2006; Thai et al., 2004). The high specificity is thought to be due to the use of eight specific primers. The process may take place at 65°C in a single tube. With the addition of a reverse transcriptase enzyme, RNA templates may also be targeted. Quantitative real time LAMP was introduced in 2004 for real time detection of genomic material with the introduction of fluorescent dyes (Zhang et al., 2014). One of the main disadvantages of LAMP assays is the increased difficulty in primer design. The higher number of primers required can be limiting for targets with smaller genomes that are notoriously variable, like a number of the foodborne viruses that will be discussed. Further, dimer formation can produce false positive results in many cases. A few factors can affect the limits of detection and sensitivity in LAMP assays, including time, temperature and MgSO_4_ concentration (Abdullahi et al., 2015).

RPA has also been the focus of numerous viral detection reports. The procedure was developed in 2006 and is currently commercialized by TwistDx, a subsidiary of Abbott Laboratories (Daher et al., 2016). The process combines isothermal recombinase-driven primer targeting of template with strand-displacement DNA synthesis. Additionally, some work suggests it can better tolerate crude DNA/RNA extraction methods compared to PCR (Moore and Jaykus, 2017a). Like LAMP, RPA can target RNA with the inclusion of a reverse transcriptase. Traditionally, primer design had been a challenge for viral targets because of the longer recommended length required for primers; however, TwistDx protocols demonstrate that PCR-length primers may be compatible with RPA, although the specific requirements for optimal primer design have not been completely elucidated. Temperatures for the RPA reaction may range from 22 to 45°C, and in most cases, RPA has been reported to have relatively high fidelity. RPA can tolerate some degree of target sequence mismatch, which may be an advantage allowing broader reactivity for viral targets with more genomic diversity (Lobato and O’Sullivan, 2018). Although RPA has been suggested to be more tolerant than PCR in the presence of known inhibitors, it still can be inhibited by high genomic DNA concentrations and high levels of sample-associated inhibitors (Lobato and O’Sullivan, 2018). RPA is more expensive than PCR for reagent costs currently, but the possibility to eliminate sample pretreatments could make up for such costs. Along with minimal sample pretreatment, RPA does not require an expensive, bulky thermocycler due to its low, constant reaction temperature. However, RPA has not yet been approved for clinical application by the FDA and may only be used for research purposes (Lobato and O’Sullivan, 2018).

## Two Bucket Syndrome: *Caliciviridae* Family, Norovirus

Human noroviruses are members of the *Norovirus* genus in the *Caliciviridae* family, and are the most common cause of known foodborne illness in the United States (Yen et al., 2011; Hall et al., 2013; Bartsch et al., 2020). Particles of norovirus are relatively small (around 37 nm in diameter), contain no lipid envelope, and have a 7.5–7.7 kilobase positive-sense single stranded RNA genome (Glass et al., 2009). The *Norovirus* genus has been divided into 10 genogroups (GI–GX), with three causing disease in humans (GI, GII, and GIV) (Chhabra et al., 2019). Noroviruses from genogroup II, genotype 4 (GII.4) cause most of the gastroenteritis cases across the globe (Moore et al., 2015). As of 2021, the CDC estimates that 900 deaths and 110,000 hospitalizations occur annually due to norovirus (Burke et al., 2021). Although the majority of cases stem from person-to-person transmission, which can occur in close (nosocomial) settings, a notable portion of transmission is associated with foods, often through food handler contamination. Norovirus gastroenteritis has been called the “two bucket disease” due to the severe vomiting and diarrhea that those infected often experience.

The first use of isothermal amplification for detection of norovirus was utilization of NASBA for Norwalk virus (GI.1), considered the lab type strain (Teunis et al., 2008). This assay had a detection limit of 10^4^ PCR units/mL of stool filtrate (Greene et al., 2003). Using the same method, GI and GII strains were evaluated, finding positive results for 13/17 different strains tested under the one broadly reactive primer set (Moore et al., 2004). Subsequently, a real-time NASBA assay was formulated that utilized broadly reactive JJV2F and COG2R primers to target GII genotypes (Lamhoujeb et al., 2009). This method reportedly displayed a superior limit of detection (a very low 0.01 PCR units) and specificity when compared to a previously established RT-qPCR assay. However, this real-time assay was only tested on GII and showcases a current challenge in norovirus detection; the need to simultaneously detect all genotypes in the GI and GII genogroups of the *Norovirus* genus accurately, specifically, and quickly. As NASBA only requires a forward and reverse primer, the work may easily be translated to PCR or other isothermal techniques, like RPA. This translation may not work so easily for other isothermal techniques that require numerous primers like LAMP; however, one group does report successful adaptation of LAMP primers for a PCR norovirus assay (Khairuddin et al., 2016).

The foundational report applying RT-LAMP for norovirus detection was published in 2006 (Fukuda et al., 2006). Sensitivities were found to be 10^2^ and 10^3^ copies/reaction for GI and GII, respectively, when using endpoint analysis by gel electrophoresis and observation of turbidity. Interestingly, the limit of detection was determined to be similar for gel electrophoresis and observation of turbidity. Further, both GI and GII designed primer sets did not exhibit cross-reactivity with a panel of other enteric viruses (rotavirus, sapovirus, adenoviruses 40 and 41, etc.), along with positive results for 6 GI and 9 GII genotypes in 90 min or less. LAMP reactions produce magnesium pyrophosphate during amplification, which can be observed as a clear white precipitate formed in the tube for endpoint detection. Similarly, nucleic acid intercalating dyes have been used for endpoint detection because they give an immediate visible readout that does not necessarily require complex equipment. However, this type of reaction does suffer in sensitivity and there is an increased possibility for false positives due to dye signal from non-specific amplification. Common types of these intercalating dyes used include hydroxynaphthol blue dye (HBD) and SYBR green I. Khairuddin et al. (2017) added SYBR I to their reaction, which resulted in a visual detection limit of 22 copies/μL NoV plasmid surrogate, with no cross-reactivity with a panel of 17 environmental bacteria. The work also demonstrated the ability of the assay to successfully detect the plasmid surrogate spiked into stream water, though more information about the amount of virus spiked and the way nucleic acid was obtained was not apparent (Khairuddin et al., 2017). HBD has been used in LAMP as well, which resulted in a 10^3^ copy/reaction detection limit from extracted stool taken from an outbreak prevalent in China (Luo et al., 2014). As the use of endpoint dyes allows for fast results, Iturriza-Gómara et al. (2008) compared the performance of the aforementioned HBD assay and a commercial RNA amplification kit. This specific kit, Loopamp RNA, has been used several times for the detection of norovirus (Fukuda et al., 2008; Iturriza-Gómara et al., 2008). Both assays resulted in comparable detection limits (10^3^ copies/reaction); however, the HBD endpoint method was found to produce signal faster by an average of 3 min. The use of HBD in LAMP is beneficial for quick detection with or without UV light (Goto et al., 2009; Liu et al., 2017), and also simplifies primer design, one of the challenges with LAMP assays. However, future work to more extensively characterize and investigate the potential for dye-based detection of these RT-LAMP assays to produce false positives is needed as presumably melting curve analysis would not be an option in a portable setting.

As stated earlier, human norovirus outbreaks have been associated with consumption of contaminated filter feeding oysters; however, detection of noroviruses in oysters remains a challenge. Jeon et al. (2017) tested the use of RT-LAMP to detect norovirus in live Pacific oysters. An assay was created to observe inhibition or irregularities in extraction of GI and GII from live Pacific oysters for both traditional and one-step RT-LAMP. Hemocytes and the exsanguinated tissue from individual oysters were individually subjected to RNA extraction and amplification. Primary testing with an RNA standard suggested the sensitivity of both the GI and GII one-step RT-PCR assays to be 4.7 × 10^2^ human norovirus GI copies/μL, while the developed real-time RT-LAMP assay had a lower detection limit of 4.7 × 10^1^ genomic copies/μL. The one step RT-LAMP assay did not cross react with a few other enteric viruses, and the respective primer sets were reactive with 5 GI and 2 GII norovirus genotypes. When using oyster RNA extract as the template, the detection limit for the RT-LAMP was found to be 2 × 10^2^ copies per 1 g of digestive gland, suggesting inhibition occurred with a reduction in sensitivity (Jeon et al., 2017).

Moore and Jaykus (2017a) reported the first application of RPA for the detection of noroviruses. Detection limits for GII.4 New Orleans were shown to be low in both kit-extracted and crudely extracted (boiled stool) RNA in RT-RPA (Moore and Jaykus, 2017a). A detection limit of 3.40 ± 0.20 log10 genomic copies per reaction for purified RNA was reported, which was about one log per reaction higher than that of an established one step RT-qPCR method, but comparable to traditional RT-PCR assays for norovirus, as well as a number of the other LAMP and NASBA assays mentioned above. One of the challenges with the designed primers given their length was the ability to more broadly react with genotypes other than GII.4, as the designed RT-RPA assay was reactive with another GII.4 strain (GII.4 Sydney), but only partially reactive with a GII.3 and not reactive with GI or a panel of other enteric viruses and bacteria. More recently, Han et al. (2020) report an RT-RPA for all GII genogroup noroviruses (including GII.4, GII.P16-GII.2, and GII.P17-GII.17) with a limit of detection of 166 copies/μL. When testing real life samples, both the RT-RPA and RT-qPCR resulted in similar positive food samples (8/20) and stool samples (10/18). However, RT-qPCR did yield 1 more positive water sample (7/17 vs. 6/17) than the RT-RPA assay. Thus, a larger number of samples in each of these matrices need to be evaluated to better understand any differences in sensitivity between the two assays, as well as further exploration of the influence of food matrices on limit of detection and specificity of the RT-RPA assay for noroviruses given its potential to be compatible with cruder sample preparation techniques. Further, the influence of different reverse transcriptases on RT-RPA assay sensitivity and ability to tolerate sample matrix-associated inhibition should be investigated in the future. Ma et al. (2018) report broad detection of murine norovirus, including strains that contained seven-point mutations from the target RPA sequence, suggesting that RPA does have some capacity to allow for even broader detection of multiple norovirus strains. More work needs to be done to understand the capacity of this technique to detect diverse norovirus strains more broadly, as well as withstand matrix-associated inhibitors present in foods. Additionally, colorimetric or dye-based detection and validation has not been reported for RT-RPA of norovirus, and further investigation into the utilization of dyes could aid in broader reactivity by removing the requirement for a lengthy fluorescent probe. Another isothermal assay that has been applied for noroviruses is a transcription-reverse transcription concerted assay (TRC assay), however, it has not been explored or reported in depth. Medici et al. (2013) evaluated this assay, with it showing specificity as well as comparable clinical sensitivity to an RT-qPCR assay when tested against 387 clinical stool samples with broad reactivity against GI and GII noroviruses, with no cross-reaction against other enteric viruses. Although these results show the potential of the TRC assay as an isothermal method for foodborne virus detection, not much work has been reported in this area and more exploration of this assay for its performance in food and environmental samples, as well as against a broader groups of genotypes would be of value (Medici et al., 2013).

## Small but Deadly: *Picornaviridae* Family

Enteroviruses are members of the *Picornaviridae* family and derive their name from the fact that they were initially isolated from the intestinal tract (Vasickova et al., 2005). These positive-sense single-stranded RNA viruses include a broad range of viruses in the *Enterovirus* genus, and cause a variety of human diseases, including the common cold, acute flaccid paralysis, acute hemorrhagic conjunctivitis, aseptic meningitis, and myocarditis. Hand, foot, and mouth disease and other diseases in this genus can be transmitted through the fecal-oral route. There are five major groups of enteroviruses: polioviruses, group A and B coxsackieviruses, echoviruses and general enteroviruses. Enteroviruses have been found to be able to persist on foods and environmental surfaces for notable periods of time under normal household storage conditions (Vasickova et al., 2005).

One of the major routes through which these viruses contaminate foods is through water. Thus, Zhao et al. (2014) reported a LAMP assay that was able to broadly detect human enteroviruses, with a focus on human enterovirus A and human enterovirus B, from water and stool samples. The detection limit was found to be 10 genomic copies/μL. The assay detected enterovirus coxsackievirus A16 (HEV-A), enterovirus 71 (HEV-A), coxsackievirus B3 (HEV-B), coxsackievirus B5 (HEV-B) and echovirus 30 (HEV-B), but not a panel of other enteric viruses, showing its specificity for HEV-A and HEV-B. Further, the developed assay showed promise when enteroviruses were inoculated into drinking water and stool samples and then extracted with a commercial viral RNA extraction kit, as all water samples tested positive, with only one of the stool samples coming back negative, possibly due to inhibitory substances in the stool (Moore and Jaykus, 2017a). Fox et al. (2002) utilized the commercial NASBA-based NucliSens^®^ kit to detect multiple enteroviruses after designing a broadly reactive primer set for detection of human enterovirus A and B. All 21 clinical samples tested that were positive for RT-PCR were also positive with the NucliSens assay (100% clinical sensitivity), including extracted cerebrospinal fluid, respiratory and stool samples. However, two stool samples that were culture-positive previously yielded negative results for RT-PCR and NucliSens assay, as well as subsequent culture, suggesting that virus had degraded in storage (Fox et al., 2002). Further, the limit of detection for the assay was found to be < 100 copies RNA per reaction with no cross reaction observed against rhinoviruses (other members of the *Picornaviridae* family). Further work with these assays utilizing cruder RNA extraction techniques would be of value for comparison to RT-qPCR assays for enteroviruses.

### Enterovirus 71

Enterovirus 71 (EV71), along with coxsackievirus A16 (below), causes hand, foot, and mouth disease, with clinical manifestations of these diseases being nearly identical. However, each of these can result in different chronic health conditions, and EV71 tends to be much more severe (Lee, 2016). While the infection is typically self-limiting, it may cause brainstem encephalitis, aseptic meningitis, and acute flaccid paralysis. Laboratory testing for the virus involves isolation of EV71 from stool, throat-swab, or cerebrospinal fluid samples.

Multiple studies have been conducted using RT-LAMP to detect EV71 in a variety of clinical samples, including nasopharyngeal swabs, stool, throat swabs, and rectal swabs. A meta-analysis by Lei et al. (2014) discusses 10 studies, with a total of 907 samples, comparing RT-LAMP to real time RT-PCR methods for EV71 detection between 2011 and 2012. The analysis suggested comparable performance of RT-LAMP methods to real time RT-PCR, as the pooled sensitivity and specificity were found to be 0.99 (95% CI: 0.97–1.00) vs. 0.97 (95% CI: 0.94–1.00), respectively. More recently, Wang et al. (2017) further evaluated this method, finding a limit of detection of 0.01 PFU per reaction. No cross-reaction of the assay was found with poliovirus 1, coxsackievirus A16, rotavirus, norovirus, sapovirus, and astrovirus (An et al., 2014). Despite the promise of this assay for EV71 detection, more evaluation in food and water samples is needed. It also should be noted that these analyses nearly all involve traditional RNA extraction techniques prior to use of the assays, which should be considered when evaluating the translatability of these assays for portable application in the future.

In trend with new technologies, Yin et al. (2018) recently reported an RT-RPA method for detection of EV71. The analytical sensitivity was found to be 3.767 log10 copies per reaction with the clinical sensitivity (95%) and specificity (100%) being comparable to a real time RT-PCR. This is particularly important as it can be difficult for these assays to differentiate EV71 with coxsackievirus A16. As mentioned for noroviruses, one of the traditional challenges with RPA-based detection—especially for viruses that have shorter genomes—is the flexibility of the assay to achieve a balance between the demand for broad reactivity with desired specificity.

### Coxsackievirus Group A

Along with causing mild hand, foot, and mouth disease, coxsackievirus infection is the most common cause of viral heart disease (Jaianand et al., 2011). Infection is shown to be more problematic in children with myocarditis and in adults with pneumonitis (Yaqing et al., 2012). There are two groups of coxsackieviruses, group A and group B, and distinguishing between both is important for proper treatment. Because of the similarities between EV71 and coxsackievirus A16, both have commonly been studied simultaneously.

Multiplex endpoint RT-LAMP has been used for simultaneous detection of EV71 strain C4 (EV71-C4) and coxsackievirus A16 using HBD dye (Nie et al., 2011). Detection limits were around 0.33 and 1.58 of a 50% tissue culture infective dose (TCID50) per reaction of EV71-C4 or CVA16, respectively. The dye successfully detected the virus in 47 extracted stool samples with 100% clinical sensitivity and no cross-reactivity with a panel of related coxsackievirus A and B strains, as well as echoviruses. However, as stated before, treatment for each disease varies and applying incorrect treatment may be detrimental to the patient; therefore, it is important to differentiate these viruses. As such, an assay exclusively targeting coxsackievirus A16 using RT-LAMP has been reported, with a detection limit of 160 copies per reaction after RNA extraction of clinical samples, which was actually about one log lower than a commercial RT-PCR kit (Yaqing et al., 2012). EV71 viruses were used with zero cross reactivity between them, suggesting strong specificity; however, other subtypes of coxsackieviruses were not tested. A similar study combined RT-LAMP technology with a lateral flow device, which had a sensitivity of 0.55 TCID50 per reaction and 100% specificity in detecting coxsackievirus A16 (Yan et al., 2015).

While coxsackievirus A16 is the most common cause of hand, foot, and mouth disease, A6 has become a major cause of outbreaks in the United States and is strongly associated with adult cases. Thus, there is a need to distinguish both for proper treatment. Wang et al. (2017) report an RT-RPA assay for coxsackievirus A6 with a detection limit of 400 copies per reaction, with 100% specificity when 234 clinical samples were tested that also included 15 coxsackievirus group A and 5 group B serotypes for which no cross-reactivity was observed. Further, the real-time RT-RPA assay showed no significant difference in sensitivity or specificity with established real-time RT-PCR assays. Reverse transcription recombinase-aided amplification assay (RT-RAA), a new isothermal amplification technology similar to RT-RPA, was also developed for coxsackievirus A6 and found to have a detection limit of 38 copies per reaction, superior to that of the aforementioned RT-RPA assay. The assay was also exhibited positive results when testing 455 clinical stool samples with superior results to a previously reported RT-PCR assay, and displayed no cross-reaction against a large panel of coxsackievirus group A and B, and echovirus samples (Yan et al., 2018).

### Coxsackievirus Group B

Coxsackievirus group B viruses can cause spastic paralysis, gastroenteritis, herpangina, pleurodynia, pericarditis, meningoencephalitis, aseptic meningitis and colds, and account for more than 25–50% of viral myocarditis cases (Gebhard et al., 1998; Monazah et al., 2017). Newborns are especially susceptible to difficult outcomes from these viruses (Kaplan et al., 1983). Jaianand et al. (2011) report an RT-LAMP detection assay for coxsackievirus group B, including B1–B5. This assay produced more positive signals than an established RT-PCR assay when tested against 31 positive stool samples, and showed no cross reaction with other enteroviruses, suggesting both favorable sensitivity and specificity. Of group B coxsackieviruses, B3 is among the most prevalent serotype, estimated to be responsible for fifty percent of viral myocarditis cases. Monazah et al. (2017) report another RT-LAMP assay for coxsackievirus B3 with a detection limit of 0.1 pg RNA per reaction with no cross-reactivity with coxsackievirus A16, echovirus, and rhinovirus. Zeinoddini et al. (2017) reported RT-LAMP that exhibited similar analytical sensitivity for coxsackievirus B3 detection (0.1 pg RNA per reaction), as stated above, and was superior to both NASBA and RT-PCR, which both exhibited at least a log higher limit of detection. The specificity of the assays was probed against a few related enteroviruses as well (Zeinoddini et al., 2017).

### Poliovirus

Polioviruses were the first viruses to be categorized as foodborne. Immunization has made wild-type strains rare (Greening and Cannon, 2016). However, detection is still important in assisting global efforts to eradicate the pathogen, as wild-type poliovirus is still endemic in two countries, as well as the current threat of circulating vaccine-derived poliovirus strains (Martinez et al., 2018; Shaghaghi et al., 2018; Alleman et al., 2020). Poliovirus Sabin strain has been used for development of an RT-LAMP assay, with a reported limit of detection of 400 copies per reaction in 50 min (Arita et al., 2009). It should be noted that this assay’s LOD is higher than other optimized RT-LAMP systems for a number of the other enteric viruses discussed above, as well as being higher than a traditional RT-qPCR assay. Given the potential threat of vaccine-derived poliovirus and wild-type polioviruses, as well as the need to utilize truly portable detection assays given the settings in which these viruses may circulate, further development and evaluation of truly portable poliovirus isothermal assays, as well as their specificity would be of public health value.

### Hepatovirus A

Hepatitis A virus (HAV) was first classified in the *Enterovirus* genus as enterovirus 72, but was subsequently given a distinct genus, *Hepatovirus* (Greening and Cannon, 2016). These viruses are further subtyped based on sequence similarity of the genes that code for the VP1 and VP3 surface proteins (Costa-Mattioli et al., 2002). Further, these viruses are not as diverse as other enteric viruses, with all seven genotypes showing 85% genetic similarity (Greening and Cannon, 2016). Being both an enteric and bloodborne pathogen, strains belonging to genotypes I and III are most predominant in humans. However, 80% of suspected cases belong to genotype I, with strain IA being the most prevalent globally (Yoneyama et al., 2007).

RT-LAMP was first introduced for the detection of hepatitis A virus by Yoneyama et al. (2007), targeting the three strains, IA, IB, and IIIB. The detection limit for all three strains was found to be 0.4–0.8 focus forming units (FFU) per reaction. Positive results could be seen via naked eye without need of a dye due to noticeable turbidity increase, similar to other LAMP reactions. Further, no cross reaction with other enteric viruses (polioviruses; norovirus genotypes I and II; sapovirus genotypes I, IV, and V; and hepatitis E virus) was observed; however, the assay was not capable of distinguishing between different HAV strains.

NASBA was used for the detection of hepatitis A on a variety of spiked agricultural samples, including wastewater, lettuce and blueberries (Jean et al., 2001). It was found that when using purified HAV target RNA in buffer, sensitivity was determined to be 0.4 ng of RNA/mL (compared to 4 ng/mL of RNA with RT-PCR) with dot blot hybridization used for visualization of the reaction. Discussion on the results of the non-extracted environmental samples can be found later in this review and in Table 2. This report further highlights the need to be cautious when extrapolating limits of detection determined against purified nucleic acid in buffer vs. those that may be observed when testing RNA extracted from clinical, food, or environmental samples.

## Do Not Wish Upon This Star: *Astroviridae* Family: Astrovirus

Astroviruses are non-enveloped, positive-sense, single-stranded RNA viruses from the *Astroviridae* family. The six points of the capsid shell give these viruses a “star like” appearance and their name. These viruses have mostly been observed to cause self-limiting gastroenteritis in animals. At least 8 serotypes known to infect humans exist, which are all antigenically different from the strains reported to infect other animals (Greening and Cannon, 2016). Astroviruses are relatively prevalent among children, causing an estimated 5–10% of gastroenteritis cases in children (Yang et al., 2014). Foodborne outbreaks of the viruses are thought to be limited, with undercooked or raw seafood and water being the most commonly associated vehicles of foodborne transmission (Greening and Cannon, 2016). Yang et al. (2014) developed an RT-LAMP assay with HBD endpoint detection for detection of astrovirus serotype I, observing a limit of detection for the assay at 36 copies/μL. When used on RNA extracted from sewage treatment plant water samples, viral RNA was found in 41.7% of samples, while an established PCR method only observed 33.3%, suggesting that this RT-LAMP assay may display better sensitivity, and/or better tolerate matrix-associated inhibitors present in sewage, than RT-PCR. Wei et al. (2013) found lower sensitivity for serotype 1 (stool samples), when compared to the previously mentioned study, with a detection limit of ∼100 RNA copies reaction. Similar to polioviruses, further development and evaluation of isothermal methods (including RT-RPA) for astroviruses should be conducted given these positive reports.

## More Than Just the Common Cold: *Adenoviridae* Family: Adenoviruses

Adenoviruses are icosahedral, non-enveloped DNA viruses, with genomes of approximately 26–45 kb in length (Kajon et al., 2019). They were first isolated from civilians and army recruits who showed symptoms of other respiratory diseases (Zaghloul, 2012). Viral infection typically can cause pneumonia, cystitis, conjunctivitis, hepatitis, myocarditis, intussusception, encephalitis and is one of the causes of the common cold (Zaghloul, 2012). While many serotypes of the virus are thought to cause upper respiratory infection, serotypes 40 and 41 are known causes of gastroenteritis. As enteric adenoviruses, they spread not only through the fecal-oral route but also through respiratory droplets. This group of viruses is estimated to be associated with 5–20% of worldwide cases of acute gastroenteritis among infants and young children (Ziros et al., 2015).

Ziros et al. (2015) report a LAMP assay utilizing SYBR Green for the endpoint detection of adenoviruses 40 and 41 in sewage samples with a process time of 60 min and 100% accuracy when compared to an established PCR method. The sensitivity of this assay for viral DNA purified from sewage samples was found to be 50–100 copies per reaction, and also exhibited specificity when tested against a panel of 12 other adenoviruses. This procedure was found to be successful in detection from extracted clinical fecal samples as well, with no false positives observed. Similarly, a multiplex RPA assay has recently been developed for detection of adenoviruses in wastewater samples with a lateral flow strip. The detection limit for viral DNA extracted from water samples was found to be 50 copies per reaction with 100% specificity and sensitivity when testing 21 samples (Rames and Macdonald, 2019). While both methods have similar detection limits and specificity for use in water, RPA takes less time and requires fewer primers. Further comparison of the two methods in more crudely processed environmental samples would be of value. Evaluation and development of these assays in a larger number of clinical and environmental samples, as well as their ability to be multiplexed, would also be of value given their prevalence.

## Isothermal Amplification and Tolerance of Matrix-Associated Inhibitors

**FIGURE 1:**
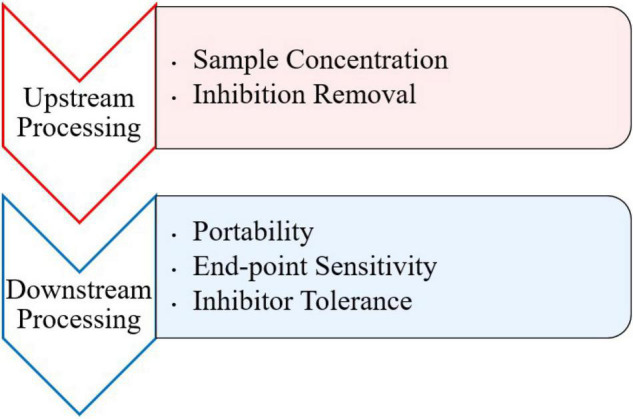
Future perspectives for foodborne viral isothermal amplification research.

Inhibitory substances present in food and environmental samples pose a problem for detection of viruses via PCR and other molecular methods. Numerous components in clinical, environmental, and food samples have been characterized as inhibitors for PCR (Thompson et al., 2014). Such inhibitors may include bile salts, complex polysaccharides, collagen, heme, humic acid, proteinases, and calcium ions (Bessetti, 2007; Suther and Moore, 2019). Although there are a variety of isothermal methods to use, most typically contain an RNA/DNA extraction step before amplification to prevent background noise and disruption of the amplification reaction from inhibitors (Stals et al., 2012). However, this requires more specialized equipment and can compromise the portability of any downstream detection assay at point of care. More recently, a few studies have observed the difference between using traditionally extracted and crudely extracted samples prior to amplification. These approaches typically involve crude extraction steps, like heating the sample to break down viral particles and release genomic nucleic acid. Furthermore, research should be conducted on effects of inhibitors during both upstream and downstream processing of isothermal assays (Figure 1).

### Clinical Samples

Stool is commonly contaminated with foodborne viral particles and rapid detection plays a critical role in prevention of outbreaks. Moore and Jaykus (2017a) investigated the direct boiling of 20 and 2% fecal suspensions of norovirus GII.4 New Orleans with RT-RPA and RT-qPCR assays. The RT-RPA assay displayed a higher positivity rate for 20% stool (61%) compared to RT-qPCR (18%), and 61 vs. 58% positivity for 2% stool; taken together, this suggests that RT-RPA may exhibit a higher tolerance of inhibitors present in stool compared to real time RT-PCR.

In addition to stool, other reports suggest higher tolerance of inhibitors associated with different clinical samples for isothermal assays compared to PCR. Nie et al. (2012) tested 145 nasopharyngeal swabs using RT-PCR and RT-LAMP. The assay demonstrated a sensitivity and specificity of 90.3 and 100%, respectively, using RT-PCR and 86.83 and 100% when using RT-LAMP. Direct RT-LAMP of EV71 on nasopharyngeal swabs that were heat-treated displayed a detection limit of 0.8 TCID50/μL (Nie et al., 2012). As opposed to the reports for stool above, this suggests that real time RT-PCR may have better tolerance than RT-LAMP when using nasopharyngeal samples with potential inhibitors. RT-LAMP has also been used for HEV in crudely processed animal tissue culture samples (Arita et al., 2009). Echovirus 11 and EV71 strains were also directly detected from crudely processed animal tissue culture samples, with the assay displaying a sensitivity of 28,000 copies and 7,400–13,000 copies per 12.5 μL, respectively (Arita et al., 2009).

### Water

As previously stated, water is a vector for several enteric viruses. Untreated metropolitan wastewater can contain a variety of contaminants, including bacteria, chemicals, human and agriculture biological waste, and pollution. Sewage samples processed without extraction (heated at 100°C for 5 min) were tested by Ziros et al. (2015) for adenovirus 40 and 41 using LAMP. The assay was able to accurately detect 93.75% (15/16) of screened urban sewage samples. Jean et al. (2001) also tested crudely processed sewage samples for HAV strain HM-175 using NASBA. Spiking 1,000 HAV PFU/μL (before treatment) into unprocessed raw wastewater, wastewater after aerobic digestion with activated sludge, and wastewater after aerobic digestion and UV treatment were tested using a NASBA assay with dot blot hybridization. Positive signals were observed in all samples; however, a weaker signal was obtained in the unprocessed raw wastewater, suggesting some level of matrix inhibition.

### Food

Rapid and portable detection of microbial pathogens in foods remains a challenge, and this is particularly the case for viruses, which cannot easily or feasibly be enriched for routine food and environmental testing. Surprisingly, few reports exist analyzing and comparing the performance of isothermal assays for viral detection in crudely treated food or food concentrate samples. Currently, a few papers on the amplification of foodborne bacteria have been published (Santiago-Felipe et al., 2015; Choi et al., 2016; Kim and Lee, 2016; Szántó-Egész et al., 2016; Gao et al., 2017). A smaller study was conducted on lettuce and blueberries inoculated with HAV strain HM-175 using NASBA, in which samples were spotted with 10^5^ HAV PFU/μL and eluate from the samples was heat treated for extraction. All eluates showed a recovery of 80% (Jean et al., 2001). In all, it appears that many isothermal amplification methods show promise for use in crudely processed samples, but much more work is needed, especially in food and environmental samples and the effect of residual matrix-associated compounds on the sensitivity and specificity of these assays.

## Discussion

Numerous isothermal amplification techniques have been reported for foodborne and enteric viruses. Of these, NASBA was the subject of much of the first series of investigation, but has quickly been replaced by LAMP with an increasing amount of focus. However, RPA has most recently been the subject of much focus for isothermal amplification of foodborne and enteric viruses, likely due in part to its shorter reaction time and reduced number of required primers. However, the use of LAMP’s several primers for specificity may be useful for viruses that are less conserved, like HAV. The use of broadly reactive and serotype-specific primers should be accurately designed in each instance and utilized based on situation. Multiple isothermal assays for a number of these viruses show promise, with comparable sensitivities to real time PCR-based methods, with less time to result and more portability in instrumentation. Further, some reports suggest that such isothermal methods may have potential to better tolerate sample matrix-associated inhibitory substances, meaning more crude nucleic acid extraction techniques may be able to be utilized to further realize the true portability of these downstream isothermal detection techniques.

However, more work needs to be conducted on the degree to which these assays can tolerate inhibitory substances from food and environmental samples, as well as the use of endpoint dyes in place of costly fluorescent probes. A fast, accurate, “suitcase” diagnostic is within the realm of possibility for isothermal amplification. However, more work is needed for detection of these viruses from food and environmental samples—including upstream sample concentration steps—as the level of viruses in these samples is often low. As further work is conducted for improved, portable upstream sample concentration and purification techniques is conducted, the need for identification of downstream detection techniques that can maintain their sensitivity in the presence of residual food and environmentally associated inhibitors is needed. In all, the collected reports discussed here reveal the promise of isothermal amplification techniques for foodborne viruses given their rapidity, portability, sensitivity, and specificity; however, more research and further development of these techniques is needed to better realize their utilization for routine detection of these viruses in food and environmental samples.

## Author Contributions

CS and MM: conceptualization. CS: writing—original draft preparation. CS, SS, YZ, and MM: writing—review and editing. MM: funding acquisition. All authors have read and agreed to the published version of the manuscript.

## Conflict of Interest

The authors declare that the research was conducted in the absence of any commercial or financial relationships that could be construed as a potential conflict of interest.

## Publisher’s Note

All claims expressed in this article are solely those of the authors and do not necessarily represent those of their affiliated organizations, or those of the publisher, the editors and the reviewers. Any product that may be evaluated in this article, or claim that may be made by its manufacturer, is not guaranteed or endorsed by the publisher.
